# Naoxintong/PPAR**γ** Signaling Inhibits Cardiac Hypertrophy via Activation of Autophagy

**DOI:** 10.1155/2017/3801976

**Published:** 2017-02-15

**Authors:** Shuping Yuan, Jianhua Jin, Lu Chen, Yongzhong Hou, Hong Wang

**Affiliations:** ^1^Institute of Life Sciences, Jiangsu University, Zhenjiang, Jiangsu 212013, China; ^2^Department of Oncology, Affiliated Wujin People's Hospital, Jiangsu University, Changzhou 212017, China; ^3^Tianjin State Key Laboratory of Modern Chinese Medicine, Tianjin University of Traditional Chinese Medicine, 312 Anshanxi Road, Nankai District, Tianjin 300193, China; ^4^Tianjin Key Laboratory of Traditional Chinese Medicine Pharmacology, Tianjin University of Traditional Chinese Medicine, 88 Yuquan Road, Nankai District, Tianjin 300193, China; ^5^Key Laboratory of Pharmacology of Traditional Chinese Medical Formulae (Tianjin University of Traditional Chinese Medicine), Ministry of Education, Tianjin 300193, China

## Abstract

As a traditional Chinese medicine, Naoxintong capsule (NXT) has been approved by China Food and Drug Administration (CFDA), which is used for cardiocerebrovascular disease treatment. Here we found that NXT extract significantly promoted H9c2 cardiomyocyte cell autophagy involved in increased autophagy-associated gene expression leading to inhibition of mTOR signaling. Moreover, NXT extract increased PPAR*γ* protein expression and transcription activity of H9c2 cell. Consistent with this, in PPAR*γ* gene silenced H9c2 cells, NXT had no effect on autophagy and mTOR signaling. Furthermore, NXT/PPAR*γ*-mediated H9c2 autophagy led to inhibition of cardiomyocyte cell hypertrophy. These findings suggest that the extract of NXT inhibited H9c2 cardiomyocyte cell hypertrophy via PPAR*γ*-mediated cell autophagy.

## 1. Introduction

As a traditional Chinese medicine, Naoxintong capsule (NXT) has been approved by China Food and Drug Administration (CFDA, Z20025001), which is used for cardiocerebrovascular disease treatment [1, 2]. NXT is a formula that consists of sixteen traditional Chinese medicines:* Radix Paeoniae* (Chishao),* Commiphora myrrha Eng1* (Moyao),* Semen Persicae* (Taoren),* Radix paeoniae Rubra* (Chishao),* Radix Angelicae Sinensis* (Danggui),* Mulberry Twig* (Sangzhi),* Achyranthes bidentata* (Niuxi),* Rhizoma Ligustici Chuanxiong* (Chuanxiong),* Salviae miltiorrhizae radix et rhizoma* (Danshen),* Spatholobus stem* (Jixueteng),* Boswellia carteri* (Ruxiang),* Cassia Twig* (Guizhi), A*stragalus membranaceus* (Fisch.) Bge. (Huangqi), and animal medicines: Scorpio (Quanxie), Pheretima (Dilong), and* Hirudo nipponica* Whitman (Shuizhi). Other reports show that NXT can decrease atherosclerosis associated with inhibition of dendritic cell maturation and iNOS expression [3, 4]. In addition, other clinical observations suggest that NXT can decrease subsequent MACE (major adverse cardiovascular events) [5]. Moreover, NXT ethanol extract decreases H2O2-mediated cardiomyocyte cell damage [6] and the development of diabetic retinopathy [7]; however, the effect of NXT on the cardiac hypertrophy is still unclear. Cardiac hypertrophy results from an increase in protein synthesis, cell size, and thickening of the heart muscle. Cardiac hypertrophy can cause heart failure and arrhythmia. However, activation of autophagy pathway can inhibit cardiac hypertrophy [8]. Autophagy delivers cytoplasmic materials or organelles into lysosomes for degradation, which is also a progress of nutrient recycling [9]. In addition, PPARs (peroxisome-proliferator-activated receptors) are the nuclear hormone receptor including PPAR*α*, PPAR*δ*, and PPAR*γ*, which play a critical role in regulation of obesity, cardiovascular diseases, and inflammation [10–13]. Other report shows that PPAR*α* regulate autophagy [14]. Here we found that NXT/PPAR*γ* signaling inhibited H9c2 cardiomyocyte cell hypertrophy via autophagy.

## 2. Materials and Methods

### 2.1. Preparation of NXT Extract

Naoxintong capsule was obtained from Buchang Pharmaceutical Co., Ltd., China. NXT powder was incubated in 60°C H2O for 6 h and then centrifuged. The supernatant was filtered and freeze-dried.

### 2.2. Cell Culture and Treatment

H9c2 cells were cultured in DMEM supplemented with 10% fetal bovine serum (FBS, Gibco). Cells were treated with or without NXT water extraction as indicated time course.

### 2.3. Immunofluorescence

H9c2 cells were fixed for 15 min with 3.7% paraformaldehyde and washed with PBS. After that, cells were blocked with BSA for 1 h, and then cells were incubated with LC3b primary antibody and subsequently with secondary antibody (Jackson ImmunoResearch). Images were taken on a confocal microscope.

### 2.4. Luciferase Assay

H9c2 cells were transfected with PPRE3-luciferase reporter and Ptk-RL and PPAR*γ* plasmids as indicated. After 24 h, cells were treated with or without 0.5 *μ*g/mL NXT for 6 h. Cell lysates were assayed by using a Dual-Luciferase reporter assay system (Promega).

### 2.5. Cell Size Assay

After treatment with or without NXT, H9c2 cells were stained with Alexa Fluor 555 phalloidin. Immunostained cells were imaged on a fluorescence microscope. Cell surface area was quantified by using image software.

### 2.6. Real-Time PCR Analysis

Total RNA was isolated using RNeasy kit (Sangon Biotech) and assayed by using Real-Time PCR assay kit (Takara). mRNA expression was normalized against GAPDH. Fold change over control was determined according to the Ct method.

### 2.7. Western Blot

H9c2 cells were seeded in 6-well plates and cultured as mentioned above. Cells were lysed in lysis buffer containing protease inhibitors. Protein concentration in the supernatant was determined by the Pierce BCA Protein Assay Kit (Thermo). The samples were subjected to 10% SDS-PAGE, transferred to a nitrocellulose membrane, then probed by Western blot analysis with the indicated antibodies, and developed by using an ECL reagent. LC3-b antibody was purchased from Novus Biologicals. Other antibodies were purchased from Sangon Biotech.

### 2.8. Statistical Analysis

Data are expressed as mean ± SEM. Statistical comparison was carried out with Student's *t*-test or one-way analysis of variance (ANOVA).

## 3. Results

### 3.1. NXT Promotes H9c2 Cell Autophagy

**Figure 1:**
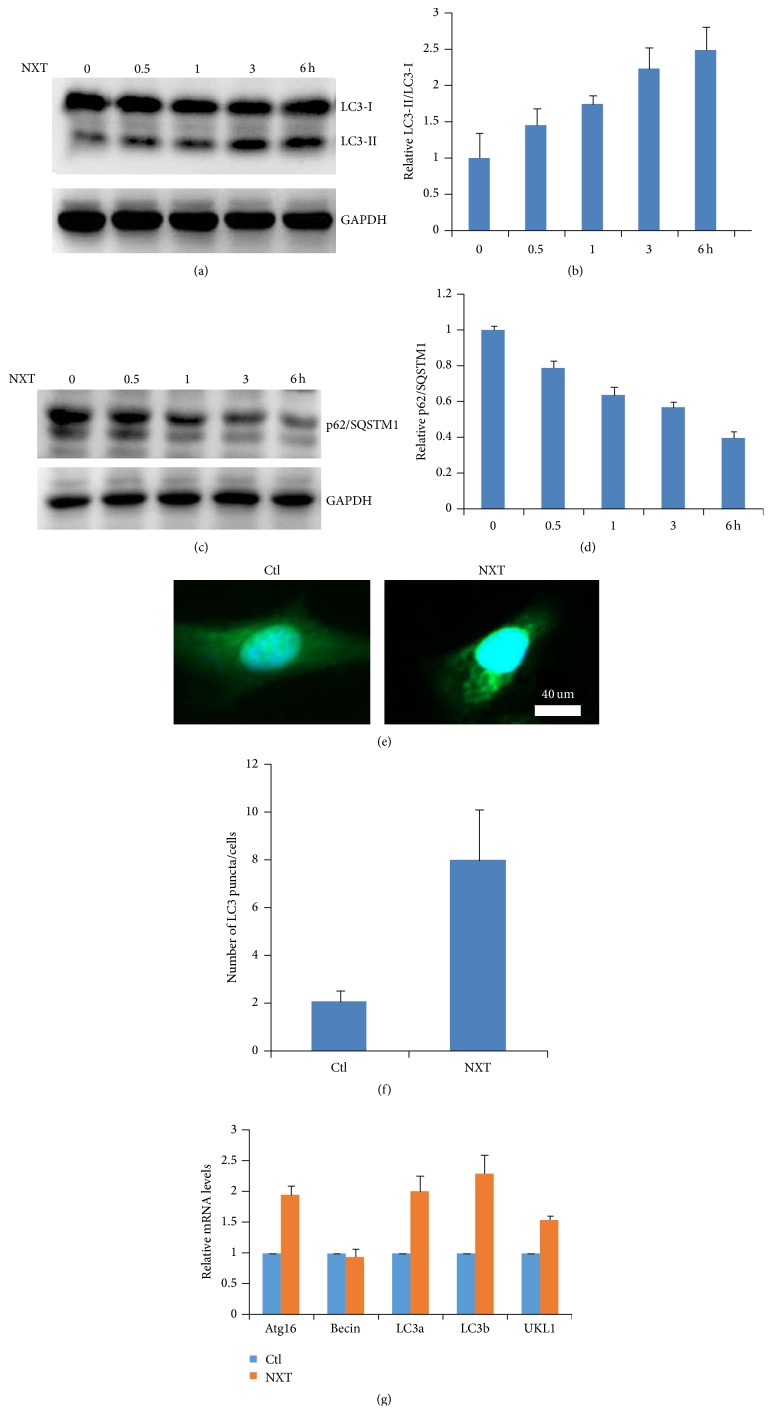
NXT promotes autophagy. (a) H9c2 cells were treated with 0.5 *μ*g/mL NXT as indicated time course. Cell lysates were subjected to Western blot. (b) Quantitation of LC3-II/LC3-I levels was shown. Results are expressed as means ± SEM (*n* = 3). (c) H9c2 cells were treated with 0.5 *μ*g/mL NXT as indicated time course. Cell lysates were subjected to Western blot. (d) Quantitation of p62/SQSTM1 levels was shown. Results are expressed as means ± SEM (*n* = 3). (e) H9c2 cells were treated with or without 0.5 *μ*g/mL NXT for 3 h. Immunofluorescence analysis was performed. (f) Quantitation of LC3 puncta was shown. Results are expressed as means ± SEM (*n* = 3). (g) H9c2 cells were treated with or without 0.5 *μ*g/mL NXT for 6 h. Autophagy-associated gene expression was assayed by qPCR. Results are expressed as means ± SEM (*n* = 3).

Figure 1(a) shows that NXT promoted H9c2 cell autophagy in a time-dependent manner, and the LC3-II/LC3-I levels were markedly increased after NXT treatment cells for 6 h. Further analysis shows that NXT time-dependent decreased the p62/SQSTM1 protein levels (Figure 1(b)). Immunofluorescence analysis shows that NXT significantly increased H9c2 cell autophagosome accumulation (Figure 1(c)), which is consistent with the Western blot results (Figure 1(a)). Autophagy-associated gene levels play an important role in activation of autophagy signaling [14]. Real-Time PCR analysis shows that NXT significantly increased the autophagy-associated gene expressions including Atg16, LC3-a, LC3b, and ULK1 (Figure 1(d)). These findings show that NXT promoted H9c2 cardiomyocyte cell autophagy involved in increased autophagy-associated gene expressions.

### 3.2. NXT Inhibits mTOR Signaling

**Figure 2:**
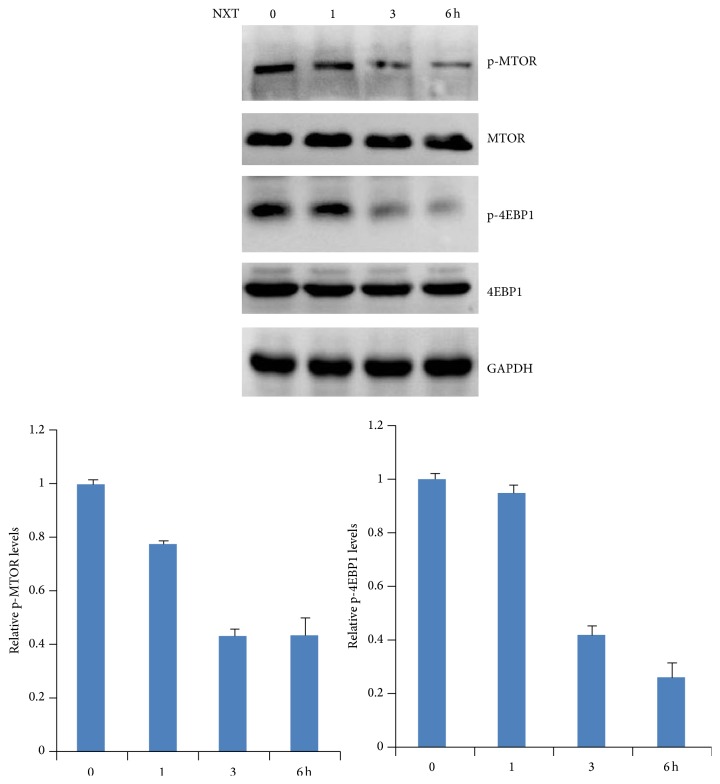
NXT inhibits mTOR signaling. H9c2 cells were treated with 0.5 *μ*g/mL NXT as indicated time course. Cell lysates were subjected to Western blot. p-mTOR or p-4EBP1 levels were quantitated. Data are expressed in triplicate from three independent experiments.

TOR (target of rapamycin) plays a critical role in promoting cell survival, proliferation, and protein synthesis; in addition, activation of TOR signaling leads to autophagy inhibition [15]. Although our results show that NXT promoted autophagy, it is still unclear whether NXT inhibited mTOR signaling. Western blot analysis shows that NXT treatment H9c2 cells significantly inhibited phosphorylation of mTOR in a time-dependent manner (Figure 2). Activation of mTOR leads to downstream signaling 4EBP1 protein phosphorylation [15]. Further analysis shows that NXT inhibited 4EBP1 phosphorylation (Figure 2). These findings show that NXT inhibited mTOR signaling pathway, which contributes to cell autophagy.

### 3.3. NXT Increases PPAR*γ* Expression

**Figure 3:**
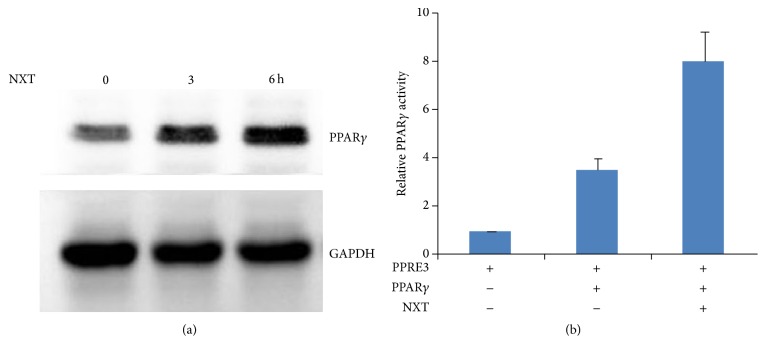
NXT increases PPAR*γ* activity. (a) H9c2 cells were treated with 0.5 *μ*g/mL NXT as indicated time course. Cell lysates were subjected to Western blot. (b) H9c2 cells were transfected with PPRE3-lu together with PPAR*γ* plasmids. Cells were treated with or without 0.5 *μ*g/mL NXT for 6 h. Dual-Luciferase assay was performed. Results are expressed as means ± SEM (*n* = 3).

Activation of PPARs can increase autophagy [14]. Further analysis shows that NXT increased PPAR*γ* protein levels (Figure 3(a)). To detect whether the expression of PPAR*γ* could enhance its transcription activity, H9c2 cells were transfected with PPRE3-luciferase reporter together with PPAR*γ* plasmids. The Dual-Luciferase assay shows that NXT significantly increased PPAR*γ* activity. These findings show that NXT increased PPAR*γ* protein levels resulting in enhancing PPAR*γ* transcription activity.

### 3.4. NXT/PPAR*γ* Signaling Promotes Autophagy

**Figure 4:**
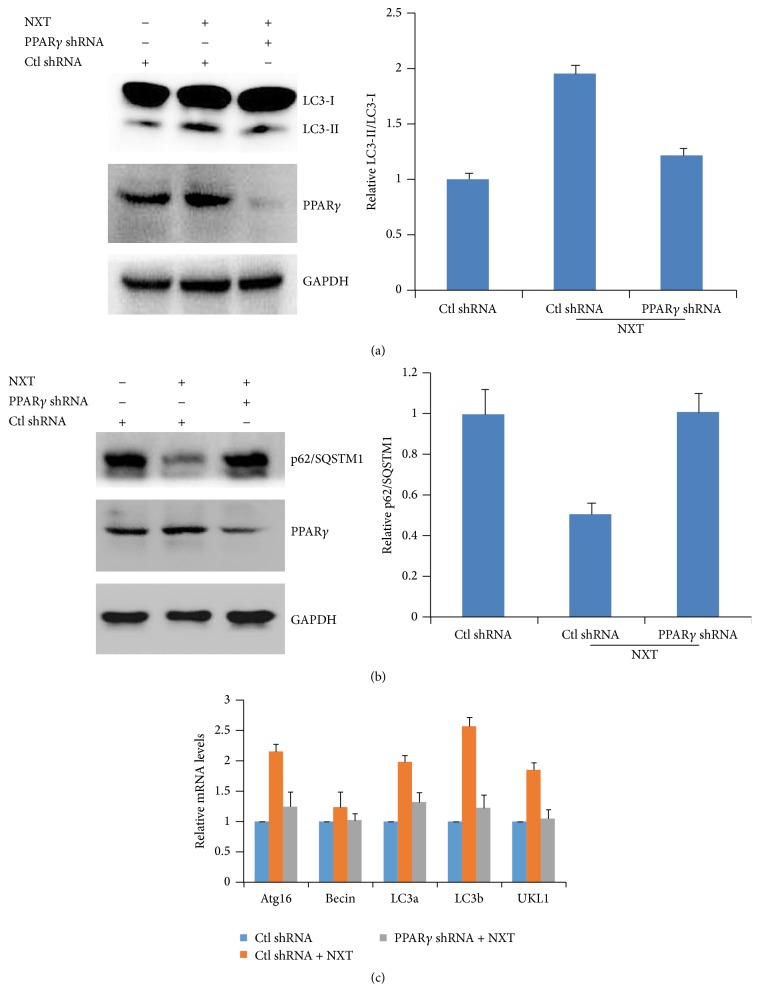
NXT/PPAR*γ* signaling mediates autophagy. (a) H9c2 cells were transfected with control shRNA or PPAR*γ* shRNA plasmids. After 24 h, cells were treated with or without 0.5 *μ*g/mL NXT for 3 h. Cell lysates were subjected to Western blot. Quantitation of LC3-II/LC3-I level was shown. Results are expressed as means ± SEM (*n* = 3). (b) H9c2 cells were transfected with control shRNA or PPAR*γ* shRNA plasmids. After 24 h, cells were treated with or without 0.5 *μ*g/mL NXT for 3 h. Cell lysates were subjected to Western blot. Quantitation of p62/SQATM1 level was shown. Results are expressed as means ± SEM (*n* = 3). (c) H9c2 cells were transfected with control shRNA or PPAR*γ* shRNA plasmids. After 24 h, cells were treated with or without 0.5 *μ*g/mL NXT for 6 h. Autophagy-associated gene expression was assayed by qPCR. Results are expressed as means ± SEM (*n* = 3).

Our data have demonstrated that NXT promoted H9c2 cardiomyocyte cell autophagy involved in increased PPAR*γ* expression. H9c2 cells were transfected with PPAR*γ* shRNA to silence PPAR*γ* expression. The results show that PPAR*γ* silenced H9c2 cells had no effect on autophagy in response to NXT stimulus (Figure 4(a)). Further analysis shows that silenced PPAR*γ* resulted in inhibition of p62/SQSTM1 degradation in response to NXT (Figure 4(b)). More importantly, silenced PPAR*γ* did not increase autophagy-associated gene expression (Figure 4(c)). These findings show that NXT/PPAR*γ* signaling increased autophagy-associated gene expression and autophagy.

### 3.5. NXT/PPAR*γ* Signaling Inhibits mTOR Signaling

**Figure 5:**
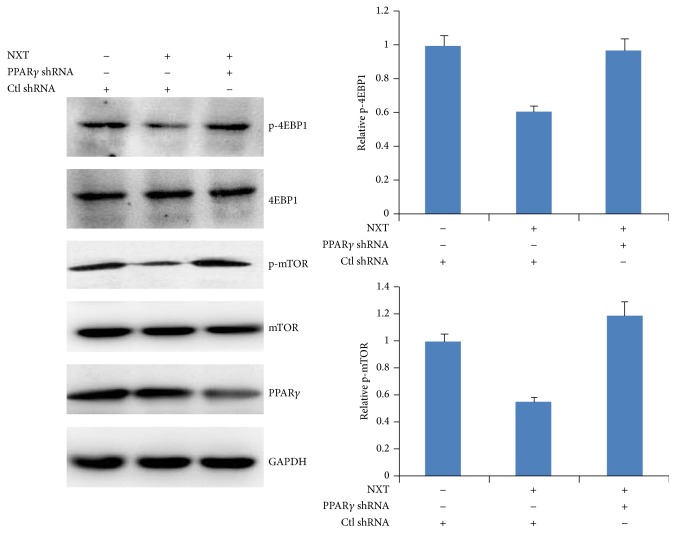
NXT/PPAR*γ* signaling inhibits mTOR activation. H9c2 cells were transfected with control shRNA or PPAR*γ* shRNA plasmids. After 24 h, cells were treated with or without 0.5 *μ*g/mL NXT for 3 h. Cell lysates were subjected to Western blot. Quantitation of p-mTOR: p-4EBP1 levels were shown. Results are expressed as means ± SEM (*n* = 3).

Activation of mTOR signaling leads to cell survival, proliferation, protein synthesis, and autophagy inhibition [15]. Our above data have demonstrated that NXT inhibited H9c2 cell mTOR signaling. We next detected whether silenced PPAR*γ* could affect mTOR signaling in response to NXT. The results show that NXT had no effect on the phosphorylation of mTOR and 4EBP1 in PPAR*γ* silenced H9c2 cells (Figure 5), suggesting that NXT/PPAR*γ* inhibited mTOR signaling.

### 3.6. NXT/PPAR*γ* Signaling Inhibits Cardiomyocyte Cell Hypertrophy

**Figure 6:**
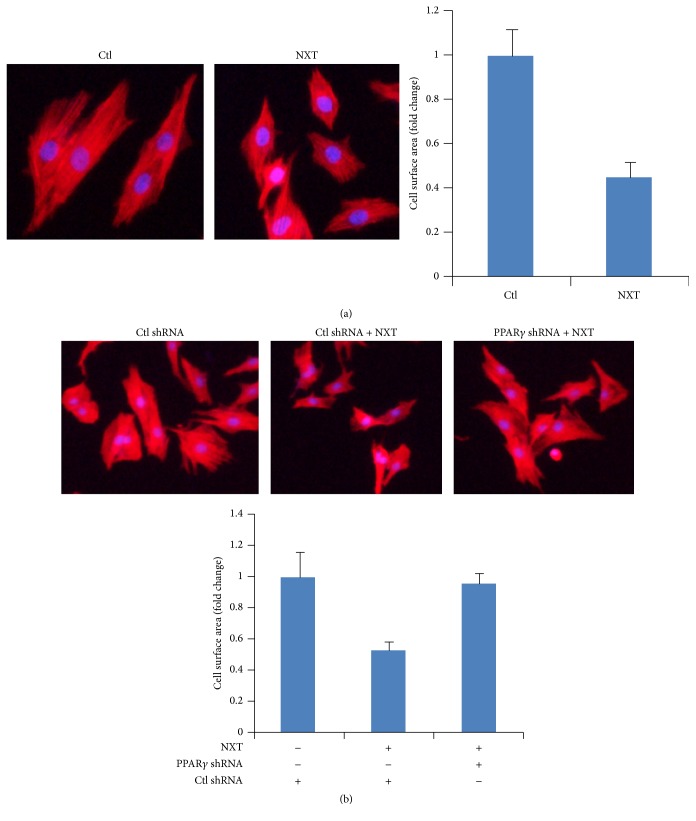
NXT/PPAR*γ* signaling inhibits cardiac hypertrophy. (a) H9c2 cells were treated with or without 0.5 *μ*g/mL NXT for 6 h. Cells were stained with Alexa Fluor 555 phalloidin. Immunostained cells were imaged on a fluorescence microscope. Cell surface area was quantified. (b) H9c2 cells were transfected with control shRNA or PPAR*γ* shRNA plasmids. After 24 h, cells were treated with or without 0.5 *μ*g/mL NXT for 6 h. Cells were stained with Alexa Fluor 555 phalloidin. Immunostained cells were imaged on a fluorescence microscope. Cell surface area was quantified.

Cardiac hypertrophy results from increased in protein synthesis and cell size. Activation of mTOR signaling promotes protein synthesis [15]. Autophagy can degrade cytoplasmic misfolded protein leading to inhibition of cardiac hypertrophy [8, 9]. Our data demonstrated that NXT/PPAR*γ* signaling inhibited autophagy and mTOR signaling. Further analysis shows that NXT inhibited H9c2 surface area by using Alexa Fluor 555 phalloidin staining (Figure 6(a)). Moreover, silenced PPAR*γ* did not reduce H9c2 cell surface area in response to NXT treatment (Figure 6(b)). These findings suggest that NXT/PPAR*γ* signaling decreased H9c2 cardiomyocyte cell hypertrophy.

## 4. Discussions

As a traditional Chinese medicine, NXT has been approved by China Food and Drug Administration (CFDA). NXT is used for treatment of cardiocerebrovascular accident [1, 2, 16]. As the Chinese herbal compound, the chemical compositions were identified by UPLC/Q-TOF-MS [17]. In addition, NXT activates PI3K-Akt pathway resulting in inhibition of oxygen-glucose deprivation/reoxygenation-induced neurons damage [18]. The combination of NXT and dual antiplatelet therapy can reduce coronary microembolization [19]. NXT suppresses atherosclerosis of the mice model [4], which is involved in reduced expression of iNOS in the vessel wall [3]. Moreover, NXT alleviates the development of diabetic retinopathy [7]. Although the ethanol extraction of NXT reduces H9c2 cell damage [6], the mechanism of NXT on the cardiomyocyte cell hypertrophy is still unclear. Here we found that NXT water extract significantly promoted H9c2 cell autophagy and autophagy-associated gene expression. Autophagy is a conserved catabolic process by delivering cytoplasmic materials or organelles into lysosomes for degradation, which is also a progress of nutrient recycling [9]. Unlike ubiquitin-proteasome system (UPS) to degrade short-lived proteins, autophagy is a bulk degradation of long-lived proteins and organelles including mitochondria, endoplasmic reticulum, nucleus, and peroxisomes [9, 20]. Autophagy can degrade misfolded or damaged proteins to maintain cellular homeostasis [9]. Other reports show that autophagy mediated regression of cardiac hypertrophy [21, 22]. Consistent with this, our data show that NXT significantly inhibited H9c2 cardiomyocyte cell hypertrophy involved in promoting autophagy. Activation of mTOR signaling pathway leads to cardiomyocyte cell protein synthesis and hypertrophy [22]. NXT inhibited mTOR signaling activation, which is benefit of inhibition of H9c2 cell hypertrophy. Although our data have demonstrated that NXT inhibited H9c2 cell hypertrophy via autophagy, the mechanism is still unclear. Peroxisome-proliferator-activated receptors (PPARs) are the nuclear hormone receptor including PPAR*α*, PPAR*δ*, and PPAR*γ*, which play a critical role in regulation of obesity, cardiovascular diseases, and inflammation [10–13]. Another report shows that PPAR*α* regulates autophagy [14]. Further analysis shows that NXT increased PPAR*γ* expression and transcription activity. Silenced PPAR*γ* inhibited NXT-mediated H9c2 cell autophagy and autophagy-associated gene expression. Consistent with this, silenced PPAR*γ* alleviated the inhibition of H9c2 cell hypertrophy in response to NXT. In addition, p62/SQSTM1 protein is autophagy cargo, which targets ubiquitinated misfolded protein for lysosome degradation to maintain cellular homeostasis [9]. Therefore, NXT/PPAR*γ* signaling-mediated p62/SQSTM1 degradation may be of benefit to inhibition of cardiomyocyte cell hypertrophy.

## 5. Conclusion

These findings suggest that NXT/PPAR*γ* signaling inhibited H9c2 cardiomyocyte cell hypertrophy via autophagy.
